# Disease-specific structural changes in thalamus and dentatorubrothalamic tract in progressive supranuclear palsy

**DOI:** 10.1007/s00234-015-1563-z

**Published:** 2015-08-08

**Authors:** Yulia Surova, Markus Nilsson, Jimmy Lätt, Björn Lampinen, Olof Lindberg, Sara Hall, Håkan Widner, Christer Nilsson, Danielle van Westen, Oskar Hansson

**Affiliations:** Department of Clinical Sciences, Lund University, Lund, Sweden; Department of Neurology, Skåne University Hospital, 221 85 Lund, Sweden; Lund University Bioimaging Center, Lund University, Lund, Sweden; Center for Medical Imaging and Physiology, Skåne University Hospital, Lund, Sweden; Department of Medical Radiation Physics, Lund University, Lund, Sweden; Department of Clinical Sciences, Malmö, Lund University, Malmö, Sweden; Memory Clinic, Skåne University Hospital, Lund, Sweden

**Keywords:** Diffusion tensor imaging, Tractography, Tract-based spatial statistics, Progressive supranuclear palsy

## Abstract

**Introduction:**

The aim of this study is to identify disease-specific changes of the thalamus, basal ganglia, pons, and midbrain in patients with progressive supranuclear palsy (PSP), Parkinson’s disease (PD), and multiple system atrophy with predominant parkinsonism (MSA-P) using diffusion tensor imaging and volumetric analysis.

**Methods:**

MRI diffusion and volumetric data were acquired in a derivation of 30 controls and 8 patients with PSP and a validation cohort comprised of controls (*n* = 21) and patients with PSP (*n* = 27), PD (*n* = 10), and MSA-P (*n* = 11). Analysis was performed using regions of interest (ROI), tract-based spatial statistic (TBSS), and tractography and results compared between diagnostic groups.

**Results:**

In the derivation cohort, we observed increased mean diffusivity (MD) in the thalamus, superior cerebellar peduncle, and the midbrain in PSP compared to controls. Furthermore, volumetric analysis showed reduced thalamic volumes in PSP. In the validation cohort, the observations of increased MD were replicated by ROI-based analysis and in the thalamus by TBSS-based analysis. Such differences were not found for patients with PD in any of the cohorts. Tractography of the dentatorubrothalamic tract (DRTT) showed increased MD in PSP patients from both cohorts compared to controls and in the validation cohort in PSP compared to PD and MSA patients. Increased MD in the thalamus and along the DRTT correlated with disease stage and motor function in PSP.

**Conclusion:**

Patients with PSP, but not PD or MSA-P, exhibit signs of structural abnormalities in the thalamus and in the DRTT. These changes are associated with disease stage and impaired motor function.

## Introduction

Progressive supranuclear palsy syndrome (PSP) is a slowly progressing fatal neurodegenerative disease with characteristic neuropathological features including the formation of tau-containing neurofibrillary tangles and neuropil threads in the basal ganglia and brainstem structures [1–3]. According to neuropathological studies, the highest level of tau-pathology in PSP is found in the substantia nigra, globus pallidus, subthalamic nucleus, red nucleus (RN), brainstem tegmentum, and dentate nucleus [4, 5]. The thalamus often also exhibits neuronal loss and gliosis, in particular, the ventral anterior (VA) and ventral lateral (VL) thalamic nuclei [6]. PSP can often be difficult to distinguish clinically from other movement disorders, e.g., Parkinson’s disease (PD), multiple system atrophy (MSA), and corticobasal degeneration (CBD), especially during early stages due to overlapping symptomatology. Patients with PSP exhibit motor symptoms, such as early development of postural instability, falls and rigidity and bradykinesia, as well as cognitive symptoms [3]. Fluorodeoxyglucose positron emission tomography (FDG-PET) has indicated reduced metabolism in the caudate nucleus, thalamus, and midbrain in PSP with thalamic hypometabolism correlating with impaired balance [7–9].

Diffusion tensor imaging (DTI) with estimation of parameters fractional anisotropy (FA) and mean diffusivity (MD) [10] has been used to study potential microstructural changes indicating neuronal pathway loss in the basal ganglia, midbrain, and pons in patients with PSP. Increased values of MD have been found in the thalamus, putamen, dorsal pons, and midbrain in PSP compared with controls [11–13]. Reduced values of FA have been observed in some white matter tracts, such as the superior cerebellar peduncles (SCP), corpus callosum, and inferior longitudinal fasciculus, as well as in the thalamus [14] with DTI parameters in the SCP differentiating PSP from other neurodegenerative diseases such as PD, MSA, and CBD [15–17].

Here, we aim at extending the previously reported findings of microstructural changes in subcortical structures by comparing diffusion parameter estimates from patients with PSP, MSA and PD, and controls and relating these findings to clinical symptoms. Two cohorts, with images acquired in different scanners with different protocols, were used in order to be able to study the reproducibility of the results, especially as the cohorts were quite modest in size. DTI parameters were quantified in the caudate head, putamen, and whole thalamus, as well as separately in some thalamic nuclei, the RN, SCP, deep cerebellar nuclei, pons, and midbrain, using region of interest (ROI) analysis, as well as in the dentatorubrothalamic tract (DRTT) [18] using tractography. Furthermore, tract-based spatial statistics (TBSS) analysis was employed to compare DTI parameters in the thalamus. Seemingly, we investigated whether the changes in DTI parameters were associated with severity of motor symptoms. We demonstrate specific diffusion changes in the thalamus and DRTT in patients with PSP that correlate with an increase in disease severity and worsening of motor function.

## Materials and methods

### Subjects

The derivation cohort included 38 subjects, 8 patients with probable PSP that were diagnosed according to the National Institute of Neurological Disorders and Stroke (NINDS) criteria [3], and 30 healthy age- and sex-matched controls. Healthy controls had no previous neurologic or psychiatric diseases. Adjunctive inclusion criteria for patients with PSP were a poor or absent response to levodopa. All patients were recruited at Skåne University Hospital. Patients and controls were evaluated using clinical assessments, among these, the Hoehn and Yahr staging scale (H&Y) [19], the Schwab and England activities of daily living scale (S&E) [20], as well as the Unified Parkinson’s Disease Rating Scale (UPDRS)-3 [21] on the on stage. The tandem gait test [22] was included to assess disturbances in balance. Cognitive function was assessed by Mini Mental State Examinations (MMSE) [23] and The Quick Test of Cognitive Speed (AQT) [24]. In addition, the PSP rating scale (PSPRS) was administered to PSP patients [25]. Patients were evaluated three times at typical intervals of 2 years, but here, we report on the PSPRS score closest to the MRI scan date (Table 1). The PSPRS comprises 28 items in six areas, the History and daily activities, the Mentation, the Bulbar, the Ocular motor, the Limb motor, and the Gate and midline with the total score ranging from 0 (normal) to 100. There was no attempt to evaluate patients off medication.

**Table 1:** Case demographics, brainstem measurements, and volumetric data for controls and cases of two cohorts

	Derivation cohort			Validation cohort				
	CTR (*n* = 30)	PSP (*n* = 8)	*P*	CTR (*n* = 21)	PD (*n* = 10)	PSP (*n* = 27)	MSA-P (*n* = 11)	*P*
Gender female:male	16:14	4:4	0.59^a^	9:12	4:6	13:14	7:4	0.68^a^
Age, years	69 (67–72)	70 (68–75)	0.38^c^	69 (65–76)	68 (59–70)	68 (65–71)	64 (56–76)	0.21^b^
Disease duration, years	n.d.	5 (4–7)		n.d.	4 (2–7)	3 (2–4)	3 (3–5)	0.54^b^
Hoehn and Yahr	0	4 (3–5)		0	2 (1–3)	3 (3–4)	3 (3–4)	≤0.00^bdf^
Schwab and England, IQL	100	55 (22–87)	≤0.00^c^	100	90 (90–100)	70 (60–80)	70 (70–80)	≤0.00^bcdefh^
Pons area, mm^2^	525 (490–546)	434 (402–527)	0.01^c^	531 (505–556)	519 (477–536)	474 (418–515)	485 (429–522)	≤0.00^bce^
Midbrain area, mm^2^	108 (97–126)	71 (46–80)	0.00^c^	117 (103–126)	116 (108–161)	63 (55–71)	113 (103–132)	≤0.00^bcdg^
Midbrain/pons ratio	0.21 (0.19–0.25)	0.16 (0.11–0.18)	<0.001^c^	0.21 (0.20–0.24)	0.24 (0.21–0.31)	0.14 (0.12–0.15)	0.22 (0.21–0.25)	≤0.00^bcdg^
Thalamus, cm^3^	9.3 ± 0.7	7.9 ± 0.5	<0.001^c^	8.7 ± 0.7	10.1 ± 1.0	8.3 ± 1.3	8.9 ± 1.1	<0.01^dh^
Caudate nucleus, cm^3^	4.4 ± 0.4	3.7 ± 0.4	0.001^c^	4.4 ± 4.5	4.7 ± 6.6	4.2 ± 0.8	4.0 ± 0.6	0.23^b^
Putamen, cm^3^	5.7 ± 0.5	4.7 ± 0.5	0.001^c^	5.5 ± 0.6	6.1 ± 0.7	4.7 ± 1.2	4.6 ± 0.7	<0.01^def^
Globus pallidus, cm^3^	2.2 ± 0.2	1.7 ± 0.4	0.001^c^	2.2 ± 0.5	2.2 ± 0.2	1.8 ± 0.6	1.8 ± 0.6	<0.01^cdf^
UPDRS motor score, IQL	0 (0–2)	43 (32–57)	<0.001^c^	n.d.	n.d.	n.d.	n.d.	
Tandem gait test, IQL	0	3 (2–3)	<0.001^c^	n.d.	n.d.	n.d.	n.d.	
MMSE score, IQL	29 (28–30)	26 (20–27)	<0.001^c^	n.d.	n.d.	n.d.	n.d.	
AQT color-form, sec	62 (57–68)	218 (90–296)	<0.001^c^	n.d.	n.d.	n.d.	n.d.	

All values expressed as medians, values in parenthesis indicate 25–75th percentiles, except volumetric measurement, which expressed as mean ± standard deviation

*Abbreviations*: *PD* Parkinson’s disease, *PSP* progressive supranuclear palsy, *MSA-P* multiple system atrophy, parkinsonian variant, *CTR* healthy controls, *MMSE* Mini Mental State Examination, *AQT* Quick Test of Cognitive Speed, *UPDRS* Unified Parkinson’s Disease Rating Scale

^a^Fisher’s exact test

^b^Kruskal-Wallis test, controls were excluded from the group comparisons of disease duration and Hoehn and Yahr scale

^c^PSP/CTR

^d^PSP/PD

^e^MSA-P/CTR

^f^MSA-P/PD

^g^MSA-P/PSP

^h^PD/CTR. Mann–Whitney *U* test

In the validation cohort, participants were recruited from the Neurology and Memory Clinics at Skåne University Hospital, Sweden, between 2008 and 2011. For the present work, 69 subjects were included, with a clinical diagnosis of probable PD (*n* = 10), probable PSP (*n* = 27), or probable MSA-P (parkinsonian variant of MSA) (*n* = 11). In addition, neurologically healthy controls were recruited (*n* = 21). Clinical diagnosis was assessed by neurologists experienced in parkinsonian disorders according to NINDS criteria [3, 26, 27]. Postmortem diagnoses were available from one PSP and one MSA-P cases. Controls, age- and sex-matched, were recruited from the Swedish population registry and had no previous neurologic or psychiatric diseases. The H&Y [19] and the S&E [20] were assessed retrospectively from medical records, without any information about on/off state during visit to doctor.

Since vascular lesions could mimic parkinsonism or be subclinical in healthy subjects, patients with anatomic MRI abnormalities and vascular lesions in midbrain and basal ganglia were ruled out by an experienced neuroradiologist who evaluated MRI scans for each subject in both cohorts.

### Data acquisition and processing

In the derivation cohort, imaging was performed using a 3 T Siemens Skyra MR scanner equipped with a 20 channel head coil. The DTI data were collected using a single-shot EPI (TE/TR 70/7500 ms/ms) sequence with diffusion encoding in 30 directions using *b* values of 0 and 1000 s/mm^2^, IPAT factor of 2, voxel size of 2 × 2 × 2 mm^3^, with an acquisition time 4 min 15 s. In the validation cohort, imaging was performed using a 3 T Philips Achieva MR scanner, equipped with an eight-channel head coil. DTI data were collected using a single-shot EPI sequence (TE/TR 90/7840 ms/ms) with diffusion encoding in 48 directions, *b* values of 0 and 800 s/mm^2^, and SENSE factor of 2.5; 60 slices were acquired with voxel size 2 × 2 × 2 mm^3^. The lower *b* value in the validation cohort was used to reduce the acquisition time. However, this will result in slightly higher values for MD [28] and thus values for MD from the derivation and validation cohorts are expected to differ. Subject motion and eddy-current correction was performed using ElastiX [29], and parameter maps were calculated using in-house developed software. For tractography, data was processed using MRtrix (Brain Research Institute, Melbourne, Australia, http://www.brain.org.au/software/) [30], including constrained spherical deconvolution (CSD) to model multiple fiber orientations in each voxel.

Volumetric data were acquired in the derivation cohort using a MPRAGE sequence with TR/TE 7/3 ms, flip angle 9°, resolution 1 × 1 × 1 mm^3^ and in validating cohort using a T1-weighted TFE sequence with TR/TE 8/4 ms, flip angle 10°, resolution 1 × 1 × 1 mm^3^.

### Analysis of diffusion parameters

**Fig. 1:**
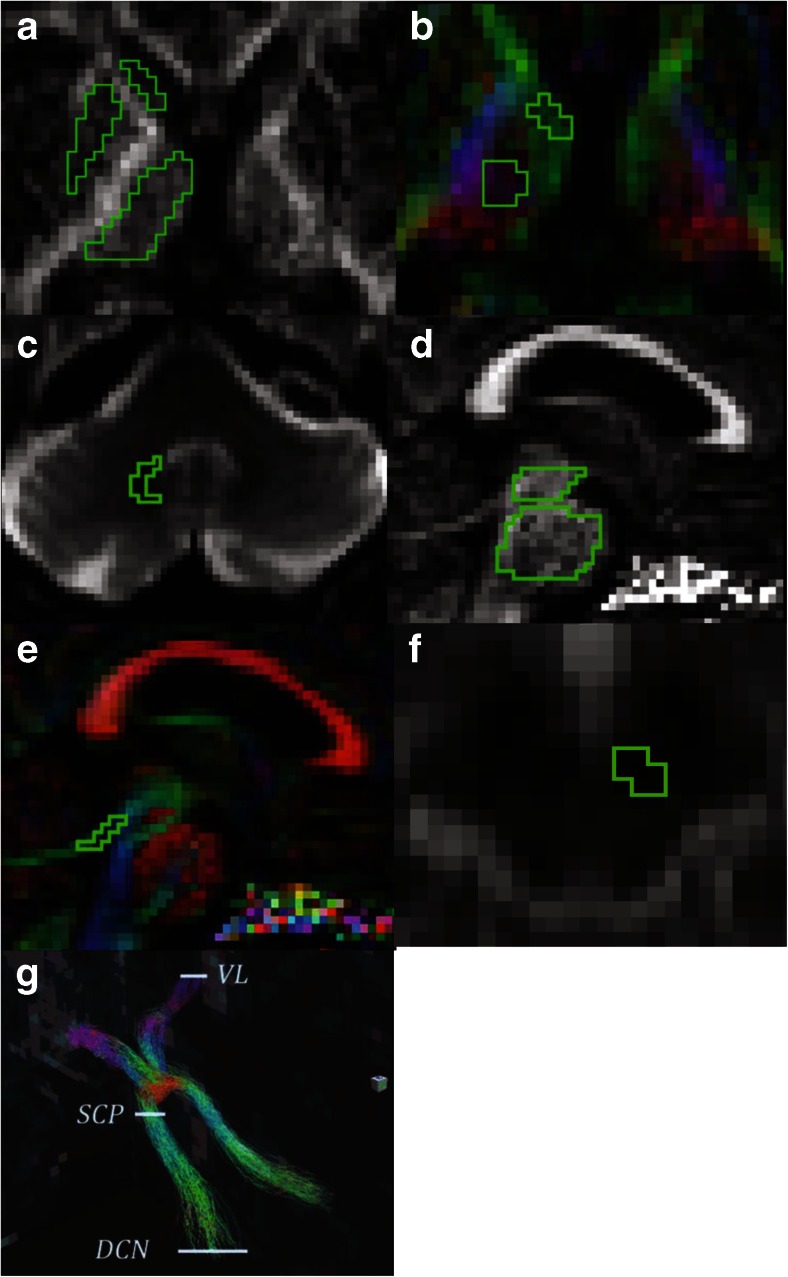
Region of interests in subcortical structures and the superior cerebellar peduncle and tractography of the dentatorubrothalamic tract. Region of interests (ROIs) are placed in **a** the putamen, caudate head, thalamus; **b** the ventral anterior and ventral lateral nuclei of the thalamus (VL), *green colored voxels* and the lateral posterior nucleus and ventral posterior nuclear complex of the thalamus, *violet colored voxels*; **c** the red nucleus; **d** the pons and midbrain; **e** superior cerebellar peduncle (SCP); **f** deep cerebellar nuclei (DCN); **g** the left and right dentatorubrothalamic tract defined by ROIs were placed in DCN, SCP, and contralateral VL
  The head of the caudate nucleus was delineated in a single slice at the level where it was most conspicuous. The thalamus and putamen were delineated in 5–8 consecutive slices at the level of the internal capsule, the thalamus adjacent to the interthalamic adhesion, and the putamen to the extreme capsule. The VA and VL nuclei of the thalamus (VAVL) were identified on FA color maps as green voxels (Fig. 1) [31, 32], in the anterior part of the lateral thalamus close to the genu of the internal capsule, while excluding the three most medial voxels that were regarded to comprise the medial dorsal nuclei. The lateral posterior nucleus (LP) and ventral posterior (VP) nuclear complex of the thalamus (LPVP) were identified on FA color maps as violet voxels (Fig. 1) [31, 32] adjacent to the posterior limb of the internal capsule and anterior to the pulvinar in red. The RN was identified as a circular area of signal hypointensity in the midbrain on non-diffusion-weighted maps and delineated in at least two consecutive axial slices. The SCP was delineated on two consecutive sagittal slices. The midbrain and the pons were delineated on five consecutive sagittal slices, with ROIs including the whole structure. The manual approach proposed by Oba et al. [33] was used to identify the boundaries of the pons and the midbrain. For each structure, the average FA and MD value from the right and left hemisphere was calculated.
- tract-based spatial statistics analysis
  TBSS (v 1.03), part of the FRMIB Software Library (FSL), was employed as a complementary analysis tool for diffusion parameters in the thalamus. Comparisons were performed in PSP vs controls in the derivation cohort and in PSP vs controls, PD and MSA-P in the validation cohort (Fig. 2) [34]. FA and MD maps were registered onto the 1 mm^3^ FMRIB58 FA template in MNI152 standard space, using the linear and nonlinear registration tools FLIRT and FNIRT [35, 36]. Before registration, the diffusion maps were masked with the FSL Brain Extraction Tool (BET) [37]. The normalized maps were skeletonized by projection onto the FMRIB58 template skeleton. Finally, the skeletonized maps were masked to include only voxels from the thalamus. The masking was done using the left and right thalamus regions in the MNI152 space Harvard-Oxford subcortical atlas, together with the requirement that the MD of the normalized maps must be less than unity in the control subjects of the derivation cohort [38].

- ROI-based analysis
  For ROI-based estimation of diffusion parameters, ROIs were outlined manually on parameter maps by one trained investigator (YS). All ROIs were outlined twice with an interval of 3 months (average intra-rater variability >0.9 for all ROIs). FA- and directionally color-encoded FA maps were used to outline all ROIs, except for the RN and deep cerebellar nuclei (DCN), where the non-diffusion-weighted map was used (Fig. 1). The ROI size was adjusted in each subject to maximize coverage of each structure, while minimizing partial volume effects from neighboring areas. Contamination from cerebrospinal fluid (CSF), which has isotropic diffusion with a high MD, was avoided by excluding voxels adjacent to the third and lateral ventricles.

**Fig. 2:**
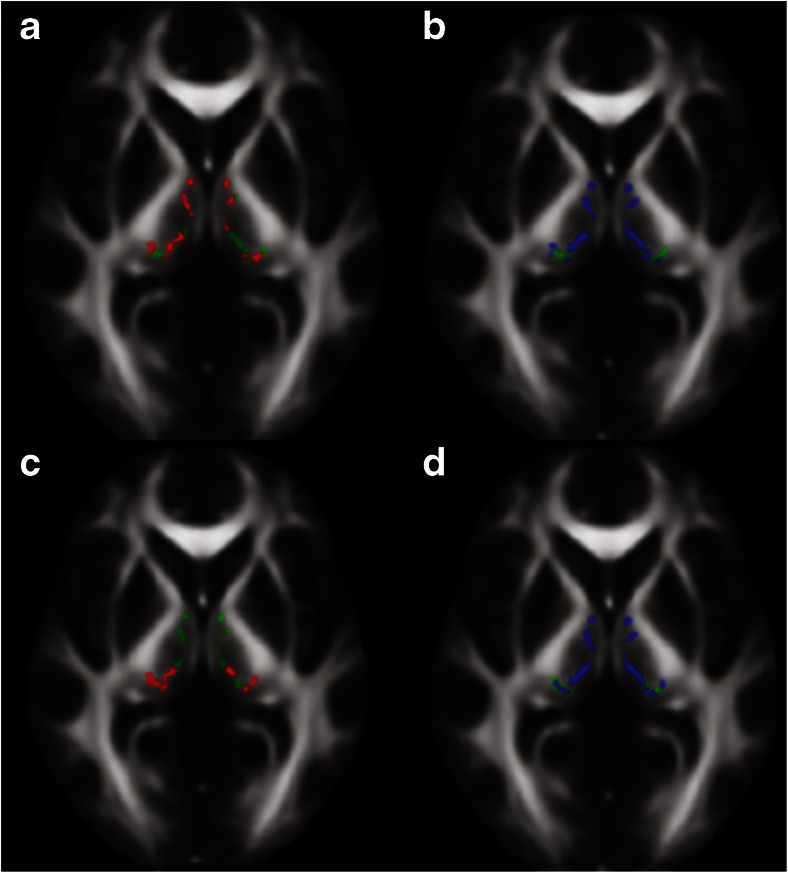
Areas in thalamus with significantly decreased fractional anisotropy and increased mean diffusivity in patients with progressive supranuclear palsy when compared to healthy controls. Results of the tract-based spatial statistics (TBSS) analysis in the thalamus, showing regions of significant decreased fractional anisotropy (*red voxels*) and increased mean diffusivity (*blue voxels*) in patients with progressive supranuclear palsy when compared to healthy controls. **a** and **b** correspond to the derivation cohort, while **c** and **d** correspond to the validation cohort. *Green voxels* are voxels on the TBSS-skeleton where no significance was found
- Tractography of the DRTT
  For probabilistic tractography of the DRTT, its inferior and superior part were constructed each using a seed ROI placed in the DCN and VAVL of the contralateral thalamus, respectively, and an include ROI in the SCP (Fig. 1). The left and right DRTT were then constructed by combining the inferior and superior DRTT into one tract, selecting fibers passing through include ROIs in these three locations, DCN, SCP, and VAVL. All tracts were visually inspected. The tractography procedure did not generate tracts in two controls from the derivation cohort (unilaterally) as well as in four PSP patients (one patient bilaterally and three unilateral) and one control (unilateral) in the validation cohort.

### Volumetric analysis

The diameter and area of the midbrain and pons, the midsagittal slice were assessed according to Oba [33]. Thus, measurements included the antero-posterior diameter of the midbrain (AP-diameter), the distance between the interpeduncular fossa, and the aqueduct in the midbrain proper without the quadrigeminal plate (IF-AQ-diameter), the surface of the pons (P-area), and the surface of the midbrain (M-area) [33, 39]. The P/M ratio was calculated as the ratio of the P-area to the M-area. Measurements were performed twice and median values were used.

Automated volumetric measurement was performed with FIRST [40]. Correction for intracranial volume (ICV) was achieved by multiplying each volume with the scaling factor that estimates the scaling between the subject’s image and standard space and that is provided by the sienax toolbox [41]. The mean volume of the right and left thalamus, caudate nucleus, putamen, and globus pallidus were used.

### Statistical analysis

Statistical analysis was performed with SPSS Statistics 20 for Windows (IBM Corporation, Somers, NY, USA). Within each cohort, differences between groups in demographic and clinical categorical variables were analyzed using the Fisher’s exact test for dichotomized data and Kruskal-Wallis test followed by Mann–Whitney *U* test for continuous data. Correlation between diffusion parameters and clinical scores was tested using Spearman correlation (Spearman’s Rho). The level of significance was set to *P* < 0.05 in the derivation and *P* < 0.008 in the validation cohorts. An adjustment for multiple comparisons between the 4 control/patient categories (i.e., 6 comparisons) was made in the validation cohort, leading to an adjusted significance level of *P* = 0.008 using Bonferroni correction. Correlation between diffusion parameters and PSPRS items was tested with linear logistic regression. Adjustment for age was performed by binary logistic regression analysis with diffusion parameter values that were significantly different between groups, based on the results of Mann–Whitney *U* test. For comparison between PSP and controls, effect sizes were computed in the form of differences in diffusional parameters group means.

To study the ability of DTI and DTT measurements to distinguish PSP from controls, MSA-P and PD in the validation cohort, receiver operator characteristic curve analysis (ROC) was performed.

Statistical processing of TBSS data was performed using Threshold-Free Cluster Enhancement of FSL Randomize (v 2.9), with 7500 permutations for the null distribution [42]. Finally, for each comparison and diffusion parameter, we computed effect sizes in the form of difference in group means in the skeletonized space in the thalamus region.

## Results

### Demographics

Demographic clinical data of patients and controls in both cohorts are reported in Table 1. Study participants of the derivation cohort were more extensively characterized compared to the validation cohort. Age, gender, and disease duration were similar in the PD, MSA-P, and PSP groups in both cohorts; however, patients with PSP and MSA-P were more disabled compared to controls as well as patients with PD.

### Diffusion parameters

- Region of interests based analysis
  In the derivation cohort, we found that patients with PSP have increased MD in the whole thalamus, the thalamic nuclei (VAVL and LPVP), and the midbrain (Table 2). Significant changes of MD (increase with 9–12 %) were found in the caudate head, thalamus, VAVL, LPVP, and midbrain. Values of FA were reduced with 12–19 % for the SCP and midbrain in PSP patients.

**Table 2:** Regional fractional anisotropy and mean diffusivity values in two cohorts

Region	Parameter	Derivation cohort		Validation cohort			
	FA	CTR	PSP	CTR	PD	PSP	MSA-P
Caudate head		0.16 (0.14–0.18)	0.18 (0.15–0.20)	0.16 (0.14–0.20)	0.17 (0.14–0.19)	0.18 (0.15–0.20)	0.16 (0.15–0.21)
Putamen		0.15 (0.14–0.17)	0.16 (0.13–0.19)	0.19 (0.17–0.20)	0.20 (0.18–0.22)	0.18 (0.17–0.20)^c^	0.22 (0.20–0.24)
Thalamus		0.29 (0.28–0.31)	0.28 (0.25–0.30)	0.32 (0.30–0.33)	0.31 (0.30–0.33)	0.30 (0.29–0.33)	0.31 (0.29–0.33)
VAVL		0.28 (0.26–0.30)	0.27 (0.24–0.30)	0.31 (0.30–0.33)	0.32 (0.29–0.34)	0.29 (0.26–0.32)^a^	0.31 (0.29–0.33)
LPVP		0.27 (0.25–0.29)	0.26 (0.24–0.28)	0.29 (0.26–0.32)	0.31 (0.29–0.33)	0.31 (0.29–0.33)	0.31 (0.29–0.35)
Red nucleus		0.45 (0.40–0.52)	0.45 (0.40–0.51)	0.54 (0.49–0.58)	0.53 (0.47–0.54)	0.50 (0.45–0.56)	0.51 (0.43–0.56)
SCP		0.66 (0.62–0.68)	0.54 (0.42–0.70)^a^	0.78 (0.75–0.82)	0.79 (0.71–0.84)	0.66 (0.60–0.71)^bc^	0.75 (0.73–0.76)
Pons		0.35 (0.34–0.38)	0.33 (0.32–0.36)	0.41 (0.39–0.46)	0.40 (0.37–0.44)	0.39 (0.36–0.42)	0.39 (0.34–0.39)
Midbrain		0.40 (0.36–0.42)	0.35 (0.30–0.38)^a^	0.45 (0.43–0.48)	0.47 (0.45–0.48)	0.40 (0.38–0.43)^abc^	0.45 (0.43–0.49)
DRTT left		0.38 (0.33–0.39)	0.33 (0.25–0.38)	0.38 (0.35–0.40)	0.39 (0.36–0.41)	0.30 (0.27–0.34)^abc^	0.37 (0.31–0.38)
DRTT right		0.37 (0.33–0.40)	0.30 (0.26–0.35)^a^	0.36 (0.33–0.37)	0.38 (0.36–0.44)	0.26 (0.24–0.29)^abc^	0.32 (0.31–0.36)^c^
	MD						
Caudate head		0.68 (0.66–0.73)	0.74 (0.63–0.79)^a^	0.79 (0.76–0.87)	0.81 (0.77–0.91)	0.82 (0.75–0.96)	0.76 (0.68–0.85)
Putamen		0.68 (0.64–0.71)	0.70 (0.66–0.76)	0.81 (0.75–0.86)	0.78 (0.76–0.86)	0.86 (0.80–0.96)^a^	0.88 (0.84–1.04)^ed^
Thalamus		0.71 (0.69–0.73)	0.79 (0.71–0.89)^a^	0.79 (0.75–0.82)	0.77 (0.76–0.81)	0.84 (0.79–0.88)^abc^	0.78 (0.77–0.80)
VAVL		0.72 (0.70–0.74)	0.81 (0.71–0.89)^a^	0.79 (0.76–0.81)	0.79 (0.77–0.83)	0.86 (0.82–0.93)^abc^	0.80 (0.76–0.83)
LPVP		0.70 (0.68–0.72)	0.77 (0.71–0.93)^a^	0.76 (0.72–0.81)	0.71 (0.70–0.75)	0.81 (0.76–0.89)^bc^	0.74 (0.74–0.75)
Red nucleus		0.48 (0.43–0.53)	0.55 (0.42–0.59)	0.53 (0.45–0.60)	0.53 0.48–0.58)	0.61 (0.55–0.68)^a^	0.61 (0.52–0.62)
SCP		0.82 (0.76–0.86)	0.85 (0.79–1.11)	0.83 (0.76–0.88)	0.82 (0.76–0.89)	0.94 (0.90–1.10)^ab^	0.92 (0.87–0.97)^d^
Pons		0.63 (0.60–0.66)	0.65 (0.62–0.70)	0.72 (0.70–0.75)	0.72 (0.70–0.75)	0.76 (0.72–0.79)^a^	0.75 (0.75–0.78)^de^
Midbrain		0.68 (0.66–0.72)	0.74 (0.71–0.81)^a^	0.74 (0.73–0.76)	0.73 (0.71–0.76)	0.83 (0.80–0.85)^abc^	0.75 (0.70–0.78)
DRTT left		0.89 (0.84–0.93)	1.02 (0.86–1.18)	1.1 (1.02–1.15)	1.03 (0.97–1.11)	1.37 (1.27–1.52)^abc^	1.21 (1.02–1.23)^c^
DRTT right		0.87 (0.80–0.93)	0.99 (0.82–1.07)^a^	1.16 (1.05–1.20)	1.06 (0.99–1.10)	1.50 (1.34–1.55)^abc^	1.22 (1.16–1.30)^c^

MD (10^–3 mm^2/s). All values expressed as medians, values in parenthesis indicate 25–75th percentiles. FA values in caudate head and putamen are low and should therefore be interpreted with caution

*Abbreviations*: *FA* fractional anisotropy, *MD* mean diffusivity, *VAVL* ventral anterior and ventral lateral nuclei of the thalamus, *LPVP* lateral posterior nucleus and ventral posterior nuclear complex of the thalamus, *SCP* superior cerebellar peduncle, *DRTT* dentatorubrothalamic tract, *PD* Parkinson’s disease, *PSP* progressive supranuclear palsy, *MSA-P* multiple system atrophy, parkinsonian variant, *CTR* healthy control

^a^PSP/CTR

^b^PD/PSP

^c^PSP/MSA-P

^d^MSA-P/CTR

**Fig. 3:**
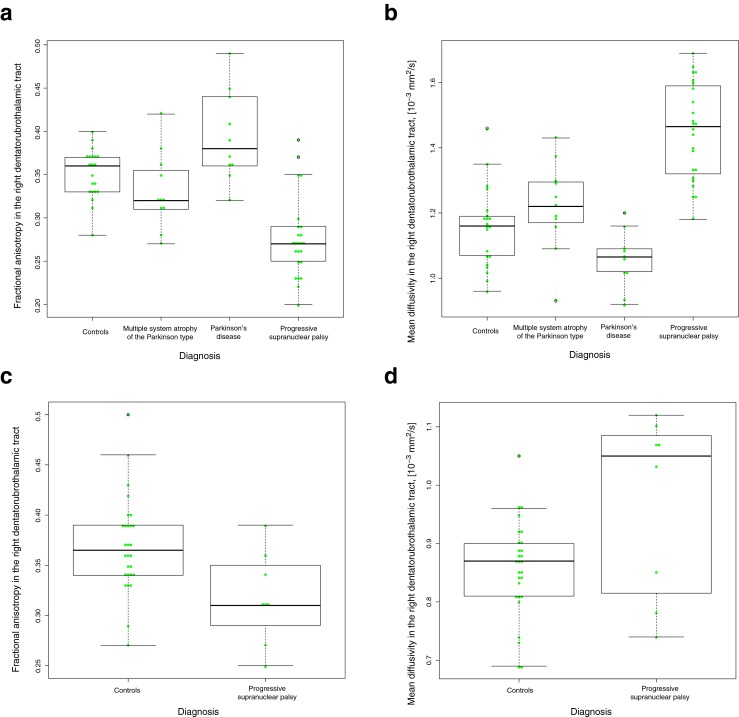
Fractional anisotropy and mean diffusivity in dentatorubrothalamic tract in derivation and validation cohorts. **a** Fractional anisotropy values in the right dentatorubrothalamic tract validation cohort. **b** Mean diffusivity values in the right dentatorubrothalamic tract validation cohort, [10^–3 mm^2/s]. **c** Fractional anisotropy values in the right dentatorubrothalamic tract derivation cohort. **d** Mean diffusivity values in the right dentatorubrothalamic tract derivation cohort, [10^–3 mm^2/s]

^e^MSA-P/PD. Mann–Whitney *U* test
  In the validation cohort, patients with PSP showed higher MD values in the whole thalamus, the VAVL nuclei of the thalamus and the midbrain compared to controls, patients with PD, and patients with MSA-P (Table 2). Patients with MSA-P displayed higher MD values in the pons than controls and patients with PD but not compared to patients with PSP (Table 2). The values of MD in PSP were increased with 6–9 % in the putamen, thalamus, VAVL, LPVP, and pons and with 12–15 % for midbrain, SCP, and red nucleus. Values of FA in PSP were reduced with 5–6 % in the putamen and VAVL, with 11–15 % in midbrain and SCP.
  In the patients with PSP, MD changes in the thalamus did not correlate with volumetric measurements in any of the cohorts.
- tract-based spatial statistics analysis
  In the derivation cohort, PSP patients showed significantly higher MD than controls in 75 % of the skeletonized voxels in the thalamus (Fig. 2). In the validation cohort, patients with PSP were found to have a higher MD in the thalamus than controls (75 % significant voxels). In the derivation cohort, a higher MD was also found in the PSP group compared to both PD (67 % significant voxels) and MSA-P (53 % significant voxels). Furthermore, a reduced FA was observed in PSP vs controls in both cohorts and in PSP vs IPD in the derivation cohort (30–50 % significant voxels).
- Tractography of the DRTT
  As many of the changes in diffusion parameters in patients with PSP observed above were localized in structures associated with the DRTT (i.e., SCP, midbrain, and ventral thalamus); probabilistic tractography of this tract was performed. In the derivation cohort, elevation in MD and reduction of FA was seen in patients with PSP, even though it only reached significance on the right side (*P* < 0.05) (Table 2 and Fig. 3c, d). Value of FA was reduced with 19 %, and value of MD was increased with 14 % for right DRTT in PSP patients. Similar to the derivation cohort, tractography of the DRTT in patients with PSP in the validation cohort exhibited reduced FA and increased MD in the DRTT on both sides when compared to both controls and patients with PD or MSA-P (Table 2 and Fig. 3a, b). The values of FA were reduced with 21–28 %, and values of MD in PSP were increased with 24–29 % in the left and right DRTT.

### Volumetric measurements

The volumes of the thalamus, the putamen, and the globus pallidus were reduced in patients with PSP in both cohorts (Table 1). The midbrain area was reduced in patients with PSP when compared to PD, MSA-P, and control subjects (Table 1). In patients with MSA-P (validation cohort only), the volumes of the putamen and the globus pallidus were reduced (Table 1). Only the thalamic volume and the midbrain area were specifically reduced in PSP.

### Correlation between clinical scales and diffusion parameters of thalamus and dentatorubrothalamic tract in progressive supranuclear palsy

In the derivation cohort, increased MD in the whole thalamus, VAVL, and LPVP correlated with increased disease stage (H&Y) and with reduced rating scores of activities of daily living (S&E) (Spearman’s Rho = −0.732–0.756, *P* < 0.05). Very similar findings were obtained in the validation cohort. There was a negative correlation between MD and disease stage (H&Y) in PSP patients in the validation cohort, similar to that in the derivation cohort, for values obtained in the whole thalamus and in the VAVL nuclei (Spearman’s Rho = 0.456 and 0.431, *P* = 0.017 and *P* = 0.025, respectively). Further, higher MD in the LPVP correlated negatively with functional measures of activity of daily living (S&E) (Spearman’s Rho = −0.467 and −0.489, *P* = 0.014 and *P* = 0.010, respectively).

The thalamic volume correlated with neither S&E nor H&Y (both cohorts).

**Fig. 4:**
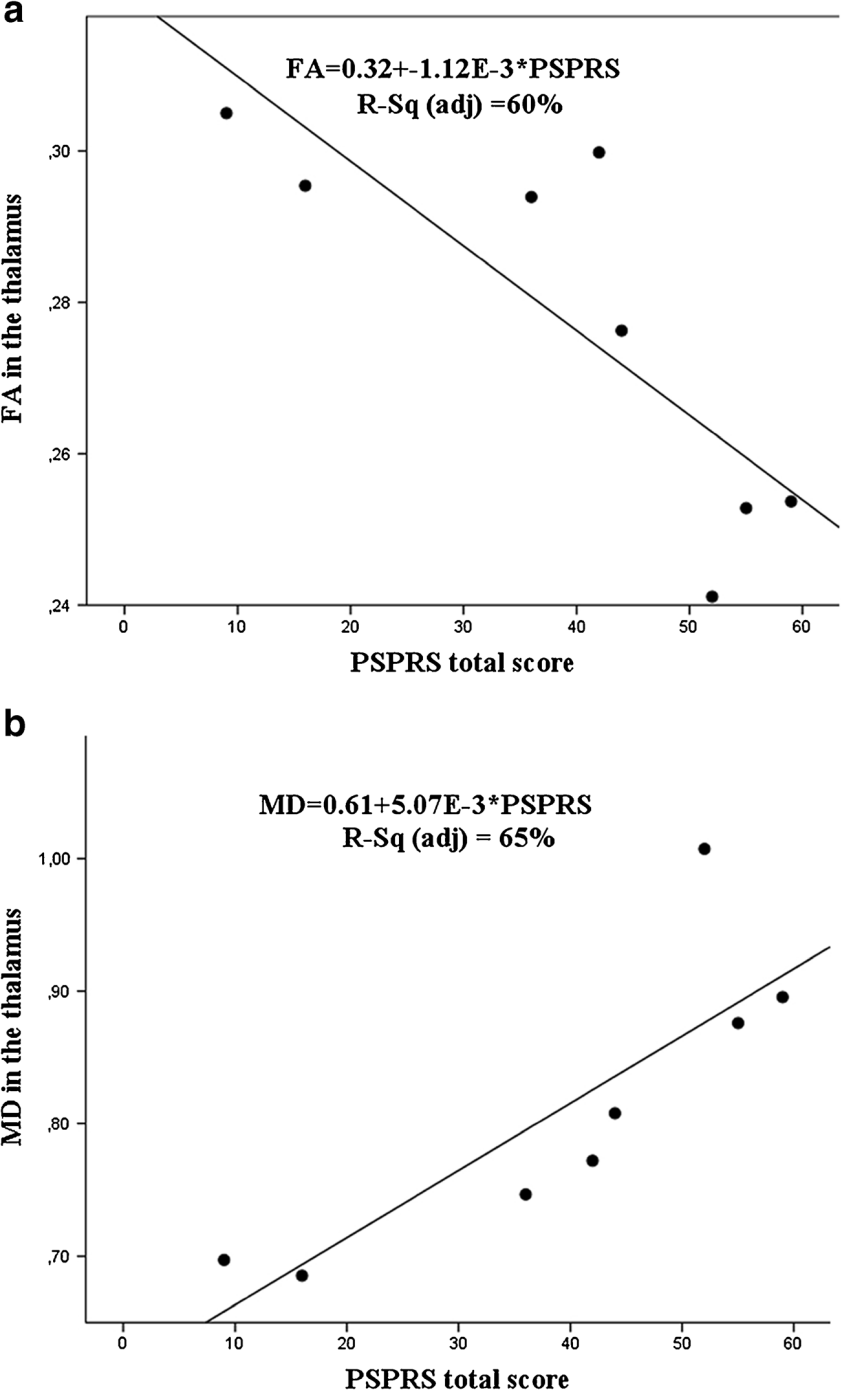
Correlation between the progressive supranuclear palsy rating score and fractional anisotropy and mean diffusivity in the thalamus for the patients with progressive supranuclear palsy in derivation cohort. **a** The regression line (*black line*) with a 95 % confidence interval (CI) shows an association between higher progressive supranuclear palsy rating score (PSPRS) and reduced fractional anisotropy in the thalamus. The correlation coefficient value is 0.81 and 60 % of variance is explained. **b** The regression line (*black line*) with a 95 % CI shows an association between higher PSPRS score and elevated mean diffusivity in the thalamus. The correlation coefficient value is 0.84 and 65 % of variance is explained

In the derivation cohort, the patients with PSP were also assessed with other clinical rating scales including UPDRS and the PSP rating scale. We found that worse motor performance (UPDRS-3) was associated with increased MD in the whole thalamus, VAVL, and LPVP (Spearman’s Rho = 0.714–0.772, *P* < 0.05) and reduced FA in the whole thalamus (Spearman’s Rho = −0.762, *P* = 0.028). Impaired balance (Tandem Gait Test) correlated with increased MD in the whole thalamus, VAVL, LPVP, but also with MD in the right DRTT (Spearman’s Rho = 0.791–0.828, *P* < 0.05). Further, we found significant correlations between diffusion changes in thalamus (whole thalamus and VAVL) and the items in the PSP rating scale (Table 3). As depicted in Fig. 4, linear regression showed highly significant positive linear correlation between MD in the thalamus in patients with PSP and the PSP rating scale total score and strong negative linear relationship between FA in the thalamus in PSP and the PSP rating scale total score.

**Table 3:** Spearman’s correlation coefficient (Rho) describing the association of diffusion tensor imaging parameters mean diffusivity and fractional anisotropy with the Progressive Supranuclear Palsy Rating Scale items scores

PSPRS items	PSPRS items scores	FA		MD	
		Thalamus	VAVL	Thalamus	VAVL
History	7.25 ± 4.62	−0.383	−0.575	0.611	0.659
Mentation	4.25 ± 3.88	−0.849‡	−0.506	0.892‡	0.892‡
Bulbar	2 ± 1.6	−0.835†	−0.933‡	0.651	0.749†
Ocular motor	8.88 ± 2.85	−0.759†	−0.313	0.699	0.723†
Limb motor	5.75 ± 1.67	−0.776†	−0.325	0.801†	0.776†
Gate and midline	11 ± 7.19	−0.735†	−0.578	0.783†	0.807†
Total	39.13 ± 18.11	−0.833†	−0.738†	0.905‡	0.929‡

PSPRS items scores expressed as mean ± standard deviation. No correction for multiple comparisons was done

*Abbreviations*: *PSPRS* Progressive Supranuclear Palsy Rating Scale, *MD* mean diffusivity, *FA* fractional anisotropy, *VAVL* ventral anterior and ventral lateral nuclei of thalamus

†*P* < 0.05, ‡*P* < 0.01, Spearman’s correlation coefficient

### Diagnostic accuracy of diffusion parameters of the midbrain, dentatorubrothalamic tract, and thalamus in progressive supranuclear palsy

Diffusion parameters from the validation cohort, that showed the most extensive differences between PSP and other diagnostic groups, were tested separately in the ROC analysis for diagnostic accuracy (Table 4).

**Table 4:** Diagnostic accuracy of mean diffusivity in the thalamus, the right dentatorubrothalamic tract, and midbrain

	ROC (95 % CI)	Cutoff MD [μm^2^/ms]	Sensitivity %	Specificity %
PSP vs controls				
Thalamus	0.77 (0.64–0.91)	0.80	74	67
DRTT R	0.95 (0.88–1.00)	1.23	96	81
Midbrain	0.86 (0.74–0.98)	0.77	85	81
PSP vs PD and MSA-P				
Thalamus	0.81 (0.68–0.93)	0.79	81	77
DRTT R	0.94 (0.87–1.0)	1.25	92	81
Midbrain	0.90 (0.88–0.99)	0.78	81	81
PSP vs PD, MSA-P, and controls				
Thalamus	0.79 (0.68–0.9)	0.8	74	71
DRTT R	0.94 (0.89–0.99)	1.24	96	79
Midbrain	0.88 (0.79–0.97)	0.77	85	79

Values refer to the validation cohort

*Abbreviations*: *PSP* progressive supranuclear palsy, *PD* Parkinson’s disease, *MSA-P* multiple system atrophy, parkinsonian variant, *DRTT R* right dentatorubrothalamic tract, *ROC* receiver operator curve analysis, *MD* mean diffusivity, *CI* confidence interval

When using the MD in the midbrain, we found that it could differentiate PSP from controls with an area under the curve (AUC) of 0.86 (Table 4), PSP from MSA-P and PD with an AUC of 0.90 (Table 4), and PSP from all groups of 0.88 (Table 4).

When using the MD in the right DRTT, we found that it could differentiate PSP from controls with an AUC of 0.95 (Table 4). Similar results were obtained when PSP separated from MSA-P and PD (Table 4) and when PSP separated from all groups (Table 4).

When using MD in the thalamus, we found that it could differentiate PSP from controls with an AUC of 0.77 (Table 4). Similar results were obtained when PSP separated from MSA-P and PD (Table 4) and when PSP separated from all groups (Table 4).

## Discussion

We have performed a study comprising two cohorts with similar demographic characteristics, comparing DTI and volumetric measurements. We derived and validated changes of MD in the thalamus, in the VAVL and LVLP thalamic nuclei and in the DRTT in PSP. Importantly, these changes were specific for PSP and correlated highly with the PSP rating scale. Furthermore, these changes were not correlated with the atrophy of thalamus and seem associated with worse motor symptoms and impaired balance.

Neurodegeneration of the thalamic nuclei in PSP has been described in a few small autopsy studies [6, 43, 44]. Tau-containing neurofilaments (NFTs) have been shown in many nuclei of the thalamus in patients with PSP. In some cases, the loss of nerve cells was severe in the dorsal part of lateral and ventral nuclei with concomitant fibrillary gliosis. Further, a comparative neuropathological study comparing cases with PD and PSP found tau-pathology in all ventral thalamic nuclei in PSP with neuronal loss most evident in the ventrolateral posterior nuclei [45]. In vivo, DTI changes in PSP have previously been shown in the thalamus when compared to controls [12, 14, 17]. Our results corroborate these findings and further suggest that changes of MD in the thalamus, in the VAVL, and LVLP thalamic nuclei are specific for PSP when compared to the other major parkinsonian disorders such as PD and MSA-P.

The present findings of changes of DTI parameters in the SCP, midbrain, and ventral thalamic nuclei in PSP suggested structural damage along the DRTT. The DRTT projects from the dentate nucleus of the cerebellum, through the SCP toward the red nucleus (with axon collaterals to this nucleus) and then proceeds superiorly to the contralateral ventral lateral and anterior nuclei of the thalamus. Degeneration of DRTT has previously been shown neuropathologically in 10 cases with PSP where degeneration and activated microglia along this tract were found [46]. Using tractography, we demonstrate reduced FA and elevated MD in the DRTT in PSP, which was confirmed in two different cohorts (Table 2; Fig. 3). Further, the quite high diagnostic accuracy obtained using the MD in the DRTT and MD in the midbrain, and to a lesser degree the MD of thalamus (Table 4), warrants further studies investigating the clinical diagnostic utility of these measures. To our knowledge, this study was first to using tractography to show changes in diffusion parameters along the DRTT in patients with PSP but not the first to show diffusion changes in regional parts of the DRTT [14].

The motor and pre-motor cortex receives thalamic inputs especially from the ventral thalamic nuclei (i.e., the “motor thalamus”) and changes in motor cortical activation are associated with the clinical features of rigidity, bradykinesia, and postural instability in both PSP and PD [45]. In this context, it is interesting to note that we found that increased MD in ventral thalamic nuclei was very consistently associated with worse motor symptoms in PSP. The ventral thalamic nuclei receive input, e.g., from the basal ganglia (globus pallidus) via the thalamic fasciculus and from the cerebellum via the DRTT. It has been suggested that the DRTT is important for postural stability. Our finding that increased MD in the DRTT is associated with impaired balance (poor performance on the tandem gait test) in patients with PSP might suggest that these changes are associated with postural instability and falls in PSP. This finding is in agreement with a previous study showing that imbalance and falls in PSP are associated with thalamic dysfunction visualized with FDG-PET, suggesting that brainstem-thalamic loops play an important role in postural imbalance and falls in PSP [9].

In the present study, we also found that the volumes of the putamen and the globus pallidus are reduced in both MSA-P and PSP, but that the volumes of the thalamus and the midbrain are selectively reduced in PSP, which is in agreement with a previous study [47]. The volumetric changes of the thalamus were not associated with clinical assessment scales in PSP, which was in sharp contrast to diffusion measures (especially MD) in the same structure, indicating that DTI more reliably detect meaningful changes in the thalamus of patients with PSP.

Our DTI results of ROI-based analysis of diffusion changes in thalamus are in agreement with the TBSS analysis of thalamus. Interestingly, our DTI results of MD changes in the infratentorial part of the DRTT in patients with PSP are in agreement with previously published results of the TBSS study [48] where patients with PSP showed white matter (WM) changes encompassing the inferior part of this tract. Our results are also in line with a previous report from another TBSS study [49] showing widespread changes in white matter tracts in both PSP and MSA patients, not found in patients with PD.

Our study has some limitations. First, the PSP group is small in the derivation cohort. However, there are previous studies demonstrating that with effect sizes above 5 %, as few as 4–7 individuals are needed when analyzing MD values in white matter structures [50]. Although the coefficients of variation were slightly higher in the structures we analyzed, the reduction in MD in PSP compared to controls in derivation cohort was up to 15 %. Given that the results were reproduced in the validation cohort, we are confident in the reliability of the results. Second, when using small cohort number, the main limitation is the risk of type II errors, not type I errors. The strength of the present study was also that the results from the derivation cohort were reproduced in the validation cohort. However, the correlations performed only in the derivation cohort (i.e., correlations with UPDRS, PSP rating scale, and tandem gate) should be interpreted with caution until validated in other cohorts. Third, manual placements of ROIs for DTI analyses can be subjective, but the ROI-based analyses of the present study resulted in high intra-rater reliability and the results were validated by the automatic TBSS analysis. Finally, clinical diagnostic criteria were used for patient collection without neuropathological confirmation in the study, thus misdiagnosis cannot be excluded. However, in two cases (one with PSP and one with MSA-P) that underwent neuropathological examination the clinical diagnoses were confirmed.

## Conclusions

We investigated disease-specific structural changes in thalamus and dentatorubrothalamic tract in PSP. In a cohort with 8 PSP patients and 30 controls, we found elevated MD in the thalamus, SCP, midbrain, and of the DRTT in patients with PSP. Increased MD in the thalamus and in the DRTT correlated with impaired motor function or balance in patients with PSP. Volumetric analysis showed reduced thalamic volumes in PSP. The DTI and volumetric findings were successfully reproduced in a validation cohort with 27 PSP patients and 21 controls. In addition, we found that most of these changes were specific to PSP and not found in patients with PD or MSA-P, which indicates that MD changes in thalamus and DRTT might be specific to impaired motor function and balance. Future studies need to be performed to examine whether changes in DTI parameters in the thalamus could be part of an MRI protocol for differential diagnosis of PSP vs PD and MSA-P. Further studies are also needed to confirm that alterations in thalamus and the DRTT are associated with impaired motor function and balance in PSP.
